# Differential DNA methylation in familial hypercholesterolemia

**DOI:** 10.1016/j.ebiom.2020.103079

**Published:** 2020-10-21

**Authors:** Laurens F. Reeskamp, Andrea Venema, Joao P.Belo Pereira, Evgeni Levin, Max Nieuwdorp, Albert K. Groen, Joep C. Defesche, Aldo Grefhorst, Peter Henneman, G.Kees Hovingh

**Affiliations:** a Department of Vascular Medicine, Amsterdam UMC, Location AMC, Meibergdreef 9, Amsterdam 1105AZ, The Netherlands; b Department of Clinical Genetics, Amsterdam UMC, Location AMC, Amsterdam, The Netherlands; c Department of Experimental Vascular Medicine, Amsterdam UMC, Location AMC, Amsterdam, The Netherlands; d HORAIZON BV, Delft, The Netherlands

**Keywords:** Familial hypercholesterolemia, *LDLR*, DNA methylation, Epigenetics

## Abstract

**Background:**

Familial hypercholesterolemia (FH) is a monogenic disorder characterized by elevated low-density lipoprotein cholesterol (LDL-C). A FH causing genetic variant in *LDLR, APOB*, or *PCSK9* is not identified in 12–60% of clinical FH patients (FH mutation-negative patients). We aimed to assess whether altered DNA methylation might be associated with FH in this latter group

**Methods:**

In this study we included 78 FH mutation-negative patients and 58 FH mutation-positive patients with a pathogenic *LDLR* variant. All patients were male, not using lipid lowering therapies and had LDL-C levels >6 mmol/L and triglyceride levels <3•5 mmol/L. DNA methylation was measured with the Infinium Methylation EPIC 850 K beadchip assay. Multiple linear regression analyses were used to explore DNA methylation differences between the two groups in genes related to lipid metabolism. A gradient boosting machine learning model was applied to investigate accumulated genome-wide differences between the two groups.

**Findings:**

Candidate gene analysis revealed one significantly hypomethylated CpG site in *CPT1A* (cg00574958) in FH mutation-negative patients, while no differences in methylation in other lipid genes were observed. The machine learning model did distinguish the two groups with a mean Area Under the Curve (AUC)±SD of 0•80±0•17 and provided two CpG sites (cg26426080 and cg11478607) in genes with a possible link to lipid metabolism (*PRDM16* and *GSTT1*).

**Interpretation:**

FH mutation-negative patients are characterized by accumulated genome wide DNA methylation differences, but not by major DNA methylation alterations in known lipid genes compared to FH mutation-positive patients.

**Funding:**

ZonMW grant (VIDI no. 016.156.445)

## 1 Introduction

Familial

**Research in Context**

**Evidence before this study**

A causal pathogenic variant in one of the Familial Hypercholesterolemia (FH) genes (i.e. *LDLR, APOB, PCSK9*) is not found in a large proportion of patients with clinical FH. We hypothesized that differential DNA methylation, a form of epigenetic regulation, contributes to the FH phenotype in these FH mutation-negative patients. We performed a PubMed search with the following terms: “Familial Hypercholesterolemia” AND “DNA methylation” and found 11 studies. None of the studies investigated the DNA methylation pattern in FH mutation-negative patients. Next, we searched PubMed with the terms ("dna methylation" OR "methylation" OR "cpg islands" OR "ewas" OR "CpG Islands"[MeSH Terms] OR "DNA Methylation"[MAJR]) AND ("ldl" OR "low-density lipoprotein") Filters: Humans. This yielded 370 articles, and in 5 of these, epigenome wide association studies showed an association between DNA methylation in multiple genes and LDL cholesterol levels. None of the studies investigated DNA methylation patterns in FH mutation-negative patients.

**Added value of this study**

This study was the first large scale study in FH mutation negative patients. In order to control for confounding due to high lipid levels we studied two unique FH patient groups: FH mutation-negative patients, and a group comprising FH mutation-positive patients. Although classical candidate gene analysis did, except for *CPT1A*, not reveal major DNA methylation differences in known lipid genes, a machine learning approach showed that FH mutation-negative patients are characterized by a different genome wide DNA methylation pattern compared to FH mutation-positive patients, with important model features for the genes *PRDM16* and *GSTT1*.

**Implications of all the available evidence**

Despite extensive sequencing efforts, a causative genetic variant is not found in a large proportion of patients with a clinical FH diagnosis. Hence efforts to find novel factors causing the FH phenotype are deemed of great relevance. Additional studies to further investigate DNA methylation and its causal role in (familial) hypercholesterolemia are warranted and might benefit from focusing on accumulation of genome-wide methylation differences instead of single gene or CpG site methylation.

Alt-text: Unlabelled box

hypercholesterolemia (FH) is a common inherited autosomal dominant disease characterized by high plasma levels of low-density lipoprotein cholesterol (LDL-C) and high risk for premature cardiovascular disease (CVD). Pathogenic variants in the genes coding the low-density lipoprotein receptor (*LDLR*), apolipoprotein B (*APOB*), and proprotein convertase subtilisin/kexin type 9 (*PCSK9*) have been shown to cause FH. However, no pathogenic variant in any of these three FH genes is identified in a large proportion of patients who are diagnosed with FH based on clinical signs and symptoms [1], fuelling an ongoing search for novel pathogenic pathways causing FH.

Differential epigenetic regulation of the genes involved in lipid metabolism may be such a factor causing FH. DNA methylation, in which a methyl group is covalently bound to the fifth carbon atom of the nucleotide cytosine when it is followed by guanine (CpG site) is the most studied form of epigenetic gene expression regulation [2]. In general, methylation of CpG sites in promoter regions of genes results in low expression of the gene, while methylation of CpG sites within the gene typically results in high expression of the gene [2].

The role of DNA methylation in lipid metabolism is relatively under investigated, but some studies have shown that DNA methylation of multiple genes is associated with plasma LDL-C as well as other lipid levels [3], [4], [5], [6]. The expression of known genes involved in LDL-C metabolism (i.e. *APOE, NPC1L1*) has been found to be regulated by CpG methylation [7,8]. Moreover, DNA methylation of multiple genes (i.e. *ABCA1, ABCG1, LIPC, PLTP, CETP*, and *LPL*) were associated with lipid traits [9], [10], [11] and coronary artery disease outcomes (i.e. *ABCA1*) [9] in patients with molecularly proven FH. However, the impact of methylation of lipid genes has not been investigated in FH patients in whom no variant in the coding region of the three major FH genes is found. In the current study we analysed the methylation pattern in patients with and without FH causing variants. A potential confounding factor is the effect of elevated lipid levels on DNA methylation itself. To overcome this issue, we compared DNA methylation in FH patients without FH causing variant (FH mutation-negative) to group of FH patients with a known pathogenic variant in *LDLR* (FH mutation-positive)*.* We not only investigated methylation differences in single genes using classical regression analysis, but also used an unbiased machine learning approach to identify whole genome differences in DNA methylation between the two groups.

## 2 Materials and methods

### 2.1 Study population

In this study we investigated DNA methylation differences between FH mutation-negative patients and FH mutation-positive patients. The Amsterdam UMC, location Academic Medical Center (AMC) in Amsterdam, is the national referral center for the genetic analysis of all Dutch patients with various forms of dyslipidaemias. For this study we analysed the DNA derived from index patients for whom the referring physician, after clinical evaluation (laboratory results, family history, and physical examination) based on national guidelines [12,13], requested molecular testing for FH causing genetic variants between 2012 and 2017. In the samples collected before 2016 (*n* = 122), molecular analysis was performed by Sanger sequencing of *LDLR, APOB*, and *PCSK9* and was followed by multiplex ligation-dependent Probe Amplification (MLPA) of *LDLR* when no pathogenic variants in these three genes were found. In samples collected from 2016 onwards (*n* = 14), a targeted next-generation sequencing (NGS) capture covering 27 lipid genes (including *LDLR, APOB*, and *PCSK9*) was used (Supplementary Table 4). Subsequent genetic cascade screening within families of index patients is done in a separate diagnostic program. These patients were not included in the current study.

The DNA of male patients was used for the current study when the patients were not a carrier for any known FH causing variant in *LDLR, APOB*, and *PCSK9* (FH mutation-negative) or had a FH causing variant in *LDLR* (FH mutation-positive). We selected patients who had plasma LDL-C levels above 6 mmol/L, which corresponds to the >99th percentile for males from all ages in The Netherlands [14]. Moreover, patients whose triglycerides levels were above 3•5 mmol/L and those who were using lipid lowering therapies (i.e. statins) at the time of DNA sampling were excluded. Females were excluded from this study because of the influence of sex differences on DNA methylation [15]. The study size was based on the availability of DNA samples of patients meeting these criteria. All included subjects gave written informed consent for re-use of their DNA samples for research into novel causes of hypercholesterolemia. The Medical Ethics Review Committee of the Amsterdam UMC, location AMC, provided a waiver for the re-use of the patients clinical data and DNA samples in the current study (reference ID: W20_246 # 20.281).

### 2.2 DNA methylation measurements

The Gentra Puregene kit was used to isolate DNA from whole blood collected in EDTA containing tubes according to standard protocols. Samples were stored at 4 °C until analysis. DNA concentrations were measured using Qubit standard methodology. DNA was treated with bisulfite using the EZ DNA Methylation kit of ZYMO® according to the standard protocol recommended by Illumina. DNA methylation of the bisulfite treated DNA was analysed with the Illumina Infinium Methylation EPIC 850 K beadchip (Illumina, California, USA) at GenomeScan (Leiden, The Netherlands). Samples of FH mutation-negative and FH mutation-positive patients were randomly assigned to different slides to avoid potential confounding batch effects.

## 3 Statistical analysis

We analysed the methylation data in a two-step approach. First, linear regression models for each CpG site were constructed to test for major difference in DNA methylation between FH mutation-positive and FH mutation-negative patients. Next, a gradient boosting machine learning technique was used to investigate unbiased subtle genome-wide DNA methylation differences between the two FH groups.

### 3.1 Quality control and normalization of methylation data

Quality control of the obtained data was performed using the R-package MethylAid (version 1.30.0), conform default settings [16]. Concordance between sex chromosome probes and self-reported sex were evaluated using principal component analyses (PCA). Next the data was normalized using the *Funnorm* function from the Minfi R package (version 1.30.0) [17]. Probes susceptible to cross-hybridization(12), probes previously described include single nucleotide polymorphisms (SNPs) with a minor allele frequency (MAF) >0.01 in either the CpG dinucleotide itself or at the position of the single base extension, and probes which included SNPs in the probe binding position were excluded (according to the Illumina manifest).

### 3.2 Candidate gene analysis

In the candidate gene analysis, CpG methylation was the dependent variable with group (FH mutation-negative or FH mutation-positive), age and leukocyte cell distributions incorporated as independent variables in this model. Leukocyte cell distributions were estimated using the obtained data according to the method of Houseman et al., resulting in information on relative cell counts of CD8+ and CD4+ *T* cells, natural killer cells, B cells, monocytes and granulocytes [18]. Quality of the epigenetic profiles was further evaluated using density plots of raw and normalized data and PCA. Correlations of the principal components one to eight with all available variables were evaluated upon entering our statistical model. For differential methylated positions (DMPs), we applied the *LMfit* function in the R package Limma (version 3.40.2). Cell distribution was determined with the R package FlowSorted.Blood.EPIC. To control for multiple testing the false discovery rate (FDR) method was used, where an FDR <0.05 was defined to be significant. We corrected for inflation using the BACON package for R (version 1.12.0) [19].

We generated four groups of genes according to the grade of impact on lipid metabolism (Supplementary Table 1). Tier 1 and 2 comprised the major (*LDLR, APOB, PCSK9*), and minor (*LDLRAP, STAP1, ABCG5, ABCG8, APOE, LIPA*) FH genes, respectively. Tier 3 comprised all genes that were shown to be significantly associated with plasma LDL-C or total cholesterol levels in a large genome wide association study [20]. Tier 4 included eighteen cytosine-guanine dinucleotide positions that have been shown to be associated with LDL-C and total cholesterol levels in previous studies [3,5,6,21]. All CpG probes within 3000 base pairs surrounding the candidate genes on either side were analysed in order to cover CpG sites in the 5` promoter region and possible downstream regulatory regions that were not annotated to a gene by the Illumina manifest.

### 3.3 Machine learning analysis

Statistical machine learning analysis was used to identify differentially methylated CpG sites that could discriminate between FH mutation-negative and FH mutation-positive subjects on a unbiased genome wide level. In brief, we used a combination of multiple gradient boosting classifiers to improve prediction accuracy [22,23]. To avoid over-fitting, we used a 5-fold stratified cross-validation over the training partition of the data (80%) while the remaining data (20%) was used as the test dataset [24]. The latter set was not used for the construction of the machine learning models. We conducted a rigorous stability selection procedure to ensure the reliability and robustness of the biomarker signatures [25]. This was repeated 50 times and Receiver Operating Characteristics (ROC) Area Under Curve (AUC) scores were computed each time and averaged for the final test ROC AUC. A permutation (randomization test) was used to evaluate statistical validity of the results [26]. In the permutation test, the outcome variable (i.e., the FH group, either FH mutation-negative or FH mutation-positive) was randomly reshuffled 1000 times while the corresponding epigenetic profiles were kept intact. By evaluating the distribution of all the results obtained in these simulations and comparing it to the outcome variable, we computed statistical significance associated with the joint panel of the selected CpG sites. To gain insight into the features that contributed the most to the model we also report relative feature importance scores for each of the CpG sites that demonstrate preferences in the model for predicting the outcome variable in the gradient boosting model. To gain insight into the biological relationship between the top features of this model and lipid metabolism, we searched for publications listed in PubMed that described a relationship between the genes identified in the top 20 contributing CpG sites and hypercholesterolemia. We used Python version 3.8 (www.python.org), with packages Numpy, Scipy and Scikits-learn for implementing the model and R version 3.5.3 (R Foundation, Vienna, Austria) for visualizations.

### 3.4 Correlation methylation and gene expression

Significantly differentially methylated CpG sites identified in the candidate gene analysis and the top 20 CpG sites that contributed the most in the machine learning model, were submitted for *in silico* validation by exploring their correlation with gene expression data in two publicly accessible liver hepatocellular carcinoma datasets; accessible via the webtools *SMART* [27] and *MEXPRESS* [28]. These datasets were based on the smaller 450 K Illumina Infinium Beadchip assay, implying that only EPIC/450 K overlapping CpG sites were investigated. Spearman's and Pearson's correlation were retrieved from both databases. Correlations between DNA-methylation and gene expression showing a P-value < 0•05 and a correlation coefficient (R) > 0•1 were suggestive to be biological relevant.

#### 3.4.1 Role of funders

The funder (ZonMW) was not involved in the design, data collection, analysis, interpretation or any other aspect of this study.

## 4 Results

Subjects for this study were diagnosed with clinical FH by the physician, who requested genetic analysis for FH in our center. The analysed cohort comprised of 78 FH mutation-negative and 58 mutation positive patients. Characteristics of the cohort are shown in table 1. FH mutation-negative patients were older (50•7 ± 12•3 *vs.* 39•1 ± 12•0 years old, *p* < 0•05 [Student's *t*-test]) had slightly lower LDL-C levels (median[IQR] 6•7 [6•4–7•2] mmol/L vs. 7•4 [6•7–8•4] mmol/L, *p* < 0•05 [Mann-Whitney U test]) and higher TG levels (1•3 [1•1–2•0] mmol/L vs*.* 1•8 [1•3–2•3] mmol/L, *p* = 0•011 [Mann-Whitney U test]) compared to the FH patients with a *LDLR* mutation.

**Table 1:** characteristics of study population.

	FH mutation-positive	FH mutation-negative	P-value*
**N**	58	78	–
**Age in years (mean (SD))**	38•1 (12•0)	50•7 (12•3)	<0•001
**Males (n (%))**	58 (100)	78 (100)	–
**Total cholesterol, mmol/L (mean (SD))**	9•6 (1•3)	9•0 (1•4)	0•022
**LDL cholesterol, mmol/L (median [IQR])**	7•4 [6•7–8•4]	6•7 [6•4–7•2]	0•001
**HDL cholesterol, mmol/L (mean (SD))**	1•3 (0•8)	1•3 (0•4)	0•668
**Triglycerides, mmol/L (median [IQR])**	1•3 [1•1–2•0]	1•8 [1•3–2•3]	0•011

SD, Standard Deviation; IQR, interquartile range; LDL, low-density lipoproteins; HDL, high-density lipoproteins.

⁎ normally distributed values (age, total cholesterol, HDL cholesterol) were compared using student's *t*-test, non-normally distributed values (LDL cholesterol and triglycerides) were compared using a Mann-Whitney U test.

### 4.1 Quality control of data

No major inflation was observed after BACON inflation correction of the data (lambda = 0•9546; Supplementary Figure 1).

### 4.2 Candidate gene analysis

**Fig. 1:**
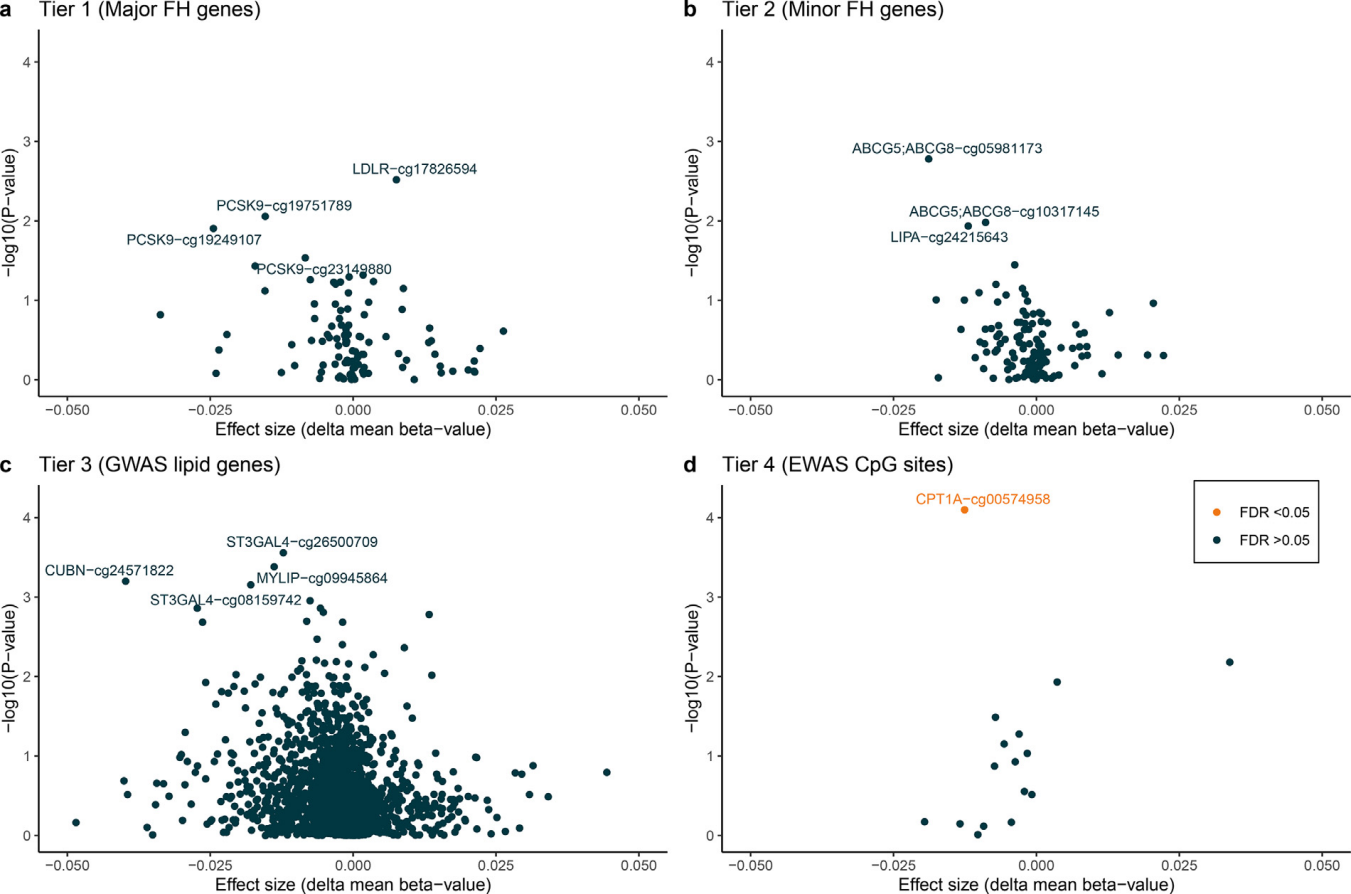
Candidate gene analysis Four tiers of genes were constructed based on literature (genes are listed in Supplementary Table 1). Shown are the difference in methylation (effect size) between FH-mutation negative and FH-mutation positive patients for the four tiers (panels A-D) Only in tier 4 (panel D), one CpG site (CPT1A-cg00574958) was significantly less methylated in FH-mutation negative patients. Significance was defined as a False Discovery rate (FDR) of <0•05. FH, Familial Hypercholesterolemia; GWAS, genome-wide association study; EWAS, epigenome-wide association study.

To investigate the association between CpG sites related to genes involved in lipid metabolism and the FH group, we performed a candidate gene analysis according to the four predefined tiers of genes (Supplementary Table 1). Tier 1 consisted of the three major FH genes: *LDLR, APOB*, and *PCSK9*. None of the studied CpG sites in these genes were significantly differently methylated in FH mutation-negative patients compared to FH-mutation positive patients (see Fig. 1, panel A). Also, in tier 2, consisting of so called “minor” FH genes, no differences between the two groups were observed (Fig. 1, panel B). Next, we investigated methylation differences in genes that were previously shown to be associated with LDL-C and total cholesterol in a large GWAS study [20]. Again, no significantly differently methylated CpG sites between FH mutation-negative and FH mutation-positive patients was found (Fig. 1, panel C). Lastly, in tier 4, consisting of CpG sites previously associated with LDL-C or total cholesterol, one CpG site (cg00574958 in the gene *CPT1A*) showed a significant 1•3% lower methylation in FH mutation-negative patients compared to FH mutation-positive patients (β −0•013, FDR = 0•001; see Fig. 1, panel D). Methylation of the identified C*PT1A* CpG site is associated with decreased expression of the *CPT1A* gene according to MEXPRESS (Supplementary Table 3) and negatively associated with triglyceride levels in our study (*r* = −0•27, *p* = 0•001 [Spearman Rank Correlation Test]).

### 4.3 Machine learning analysis

**Fig. 2:**
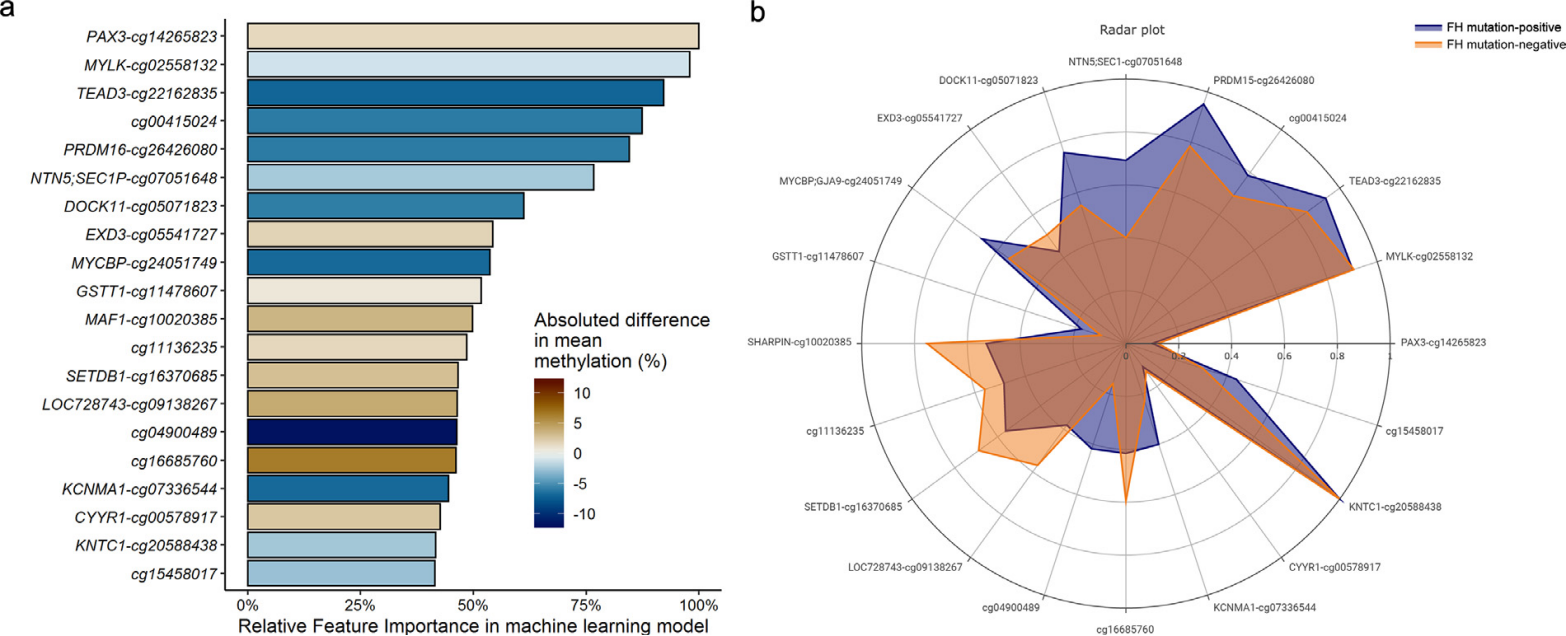
Top 20 machine learning identified CpG sites Top 20 CpG sites most contributing to the machine learning model performance, selected on relative feature importance. (A) Bar chart of top 20 CpG sites ordered from highest relative feature importance to lowest, coloured for absolute difference in mean methylation (%) in FH mutation-negative patients vs. FH mutation-positive patients. (B) Radar plot displaying top 20 CpG cites that differentiate between FH mutation-negative and FH mutation-positive patients. The axes represent the standardized mean CpG methylation levels (scaled zero-mean unit-variance).

Clearly, methylation of single genes is not likely to account for the FH phenotype in FH mutation-negative patients. To investigate whether methylation changes in multiple genes may cause the defect we applied machine learning on the whole genome methylation data set. Next, a gradient boosting machine learning analysis was applied on the whole dataset for the discovery of genome wide differences in methylation between FH mutation-positive and FH mutation-negative patients. A hierarchical structure was generated based on the effect size and the top 20 probes with the highest relative feature importance in this model are reported in Table 2 and shown in Fig. 2. Fifty percent of the top 20 CpG sites were hypermethylated with the biggest median methylation difference between the two groups for the genes *PRDM16, GSTT1*, and *LOC728743* (Fig. 2A). In contrast, *DOCK11* and *KCNMA1* were most differentially hypomethylated in FH mutation-negative compared to FH mutation-positive patients. All probes with a Relative Feature Importance >10% are listed in Supplementary Table 2.

**Table 2:** Top 20 machine learning identified CpG sites.

	CpG	Gene	Chromosome	Position^1^	Gene feature	Methylation direction in FH mutation- negative^2^	Relative Feature Importance	Protein function^3^
**1**	cg14265823	*PAX3*	chr2	223,163,326	Exon 1	Hyper	100	Paired Box 3; involved in neural development and myogenesis during fetal development.
**2**	cg02558132	*MYLK*	chr3	123,411,198	Intron 19	Hypo	97•97	Myosin light chain kinase; involved in smooth muscle contraction via phosphorylation of myosin light chains.
**3**	cg22162835	*TEAD3*	chr6	35,457,472	Intron 1	Hypo	92•2	TEA Domain Transcription Factor 3; mainly expressed in placenta and involved in transactivation of chorionic somatomammotropin-B.
**4**	cg00415024		chr20	56,044,352	Intergenic	Hypo	87•39	
**5**	cg26426080	*PRDM16*	chr1	3,039,210	Intron 1	Hypo	84•61	PR/SET Domain 16; transcriptionfactor involved brown adipose tissue differentiation.
**6**	cg07051648	*NTN5/SEC1P*	chr19	49,177,693	Intron 4 (SEC1P)	Hypo	76•65	Netrin 5; plays a role in neurogenesis, prevents motor neuro cell body migration out of the neural tube.
**7**	cg05071823	*DOCK11*	chrX	117,628,671	Intergenic	Hypo	61•17	Dedicator Of Cytokinesis 11; involved in megakaryocyte development and platelet production.
**8**	cg05541727	*EXD3*	chr9	140,277,740	Intron 2	Hyper	54•31	Exonuclease 3′−5′ Domain Containing 3; involved in RNA degradation.
**9**	cg24051749	*MYCBP*	chr1	39,340,282	Intron 1	Hypo	53•71	MYC Binding Protein; can bind to oncogenic protein C-MYC and is possibly involved in spermatogenesis
**10**	cg11478607	*GSTT1*	chr22	24,384,400	Intergenic	Hyper	51•79	Glutathione S-Transferase Theta 1; conjungates reduced glutathione to exogeneous and endogeneous hydrophobic electrophiles.
**11**	cg10020385	*MAF1*	chr8	145,159,706	Exon 1	Hyper	49•8	Repressor of RNA polymerase III transcription MAF1 homolog; involved in repression of RNA polymerase III-mediated transcription.
**12**	cg11136235		chr10	81,077,552	Intergenic	Hyper	48•55	
**13**	cg16370685	*SETDB1*	chr1	150,899,163	Intron 1	Hyper	46•59	SET Domain Bifurcated 1; regulates histone methylation, potential target for treatment in Huntington Disease
**14**	cg09138267	*LOC728743*	chr7	150,102,791	Intron 1	Hyper	46•47	Zinc Finger Protein Pseudogene
**15**	cg04900489		chr13	31,272,551	Intergenic	Hypo	46•29	
**16**	cg16685760		chrX	145,701,257	Intergenic	Hyper	46•17	
**17**	cg07336544	*KCNMA1*	chr10	79,194,347	Intron 1	Hypo	44•54	Potassium Calcium-Activated Channel Subfamily M Alpha 1; encodes alpha subunit of the MaxiK calcium-sensitive potassium channels in smooth muscle cells.
**18**	cg00578917	*CYYR1*	chr21	27,945,542	Exon 1	Hyper	42•69	Cysteine And Tyrosine Rich 1
**19**	cg20588438	*KNTC1*	chr12	123,089,881	Exon 51	Hypo	41•65	Kinetochore Associated 1; involved in proper chromosome segregation during cell division
**20**	cg15458017		chr17	9,672,274	Intergenic	Hyper	41•5	

Top 20 CpG sites sorted by relative feature importance for contribution in the machine learning model distinguishing FH mutation-negative from FH mutation-positive subjects.

1 Genomic positions as provided in human genome build – hg19.

2 Hypo- or hypermethylation in FH mutation negative group compared to FH mutation-positive group, based on direction of difference in median normalized beta's in both groups (see Supplementary Figure 2).

3 Gene names and functions (when known/available) were derived from GeneCards.org(Stelzer et al., 2016).

Most of the top 20 CpG sites were located within introns or exons of known genes, and none are located in promotor regions of genes. Of the top 20 CpG sites, five were not located in close proximity of a gene. Eleven of the top 20 CpG sites were hypomethylated in FH mutation-negative patients compared to FH mutation-positive patients. Boxplots of the methylation per top 20 CpG site per patient group are shown in Supplementary Figure 2 and their correlation with gene expression in Supplementary Table 3.

**Fig. 3:**
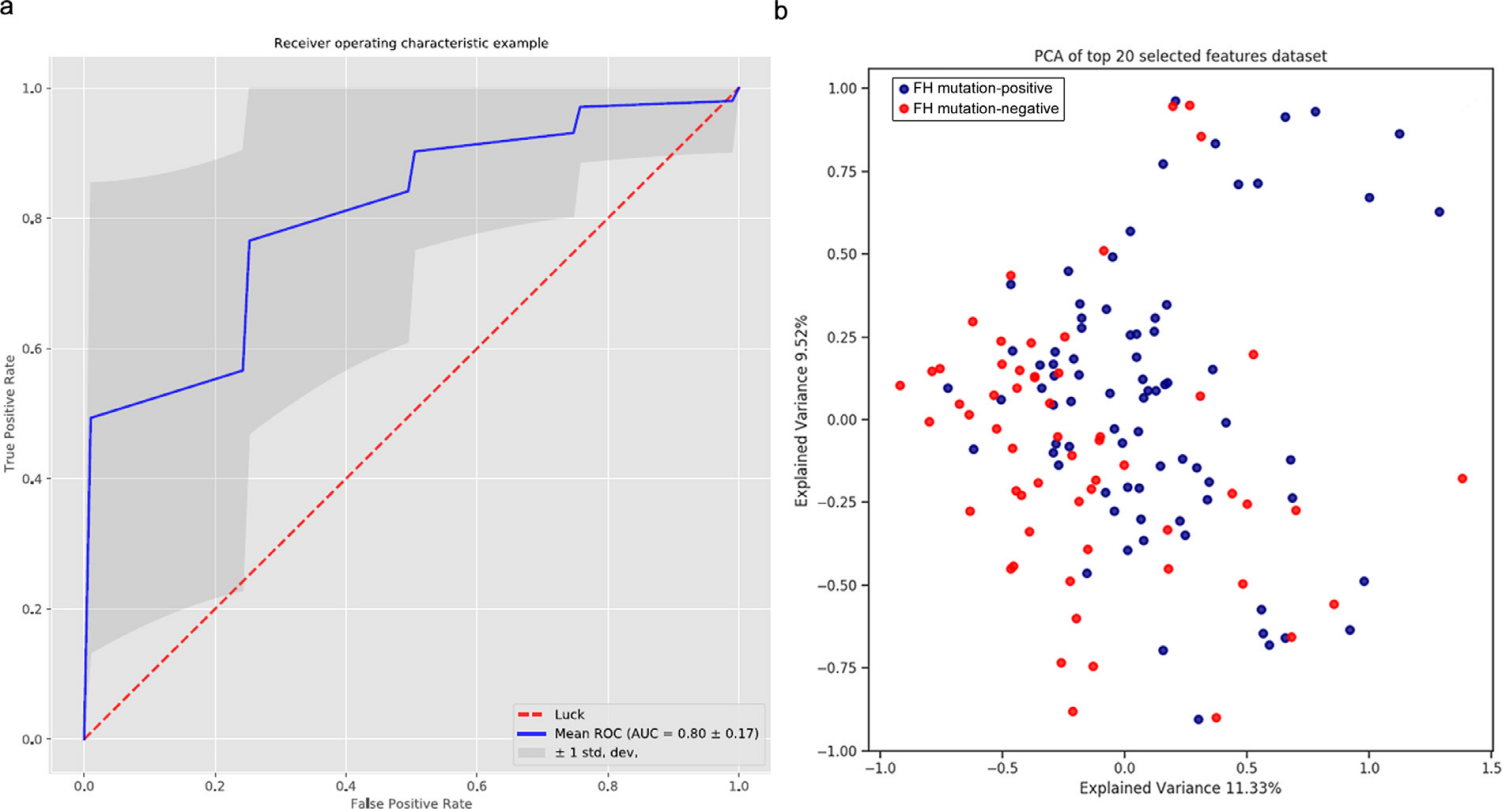
Performance of machine learning model Performance of machine learning model in distinguishing FH mutation-negative from FH mutation-positive patients. (A) ROC curve of the model. The machine learning model was able to distinguish FH mutation-positive and FH mutation-negative patients with an Area Under the Curve (AUC±SD) of 0•80±0•17. (B) Principle Component Analysis of the top 20 CpG sites with the highest relative feature importance.

The model generated by machine learning distinguishes methylations landscape in FH mutation-negative and FH mutation-positive patients with an average Area Under the Curve (AUC) of 0•80±0•17 over 50 repeat runs with different validation and test sets(Fig. 3A). A principle component analysis showed an explained variance of 11•33% for component 1 and 9•52% for component 2 (Fig. 3B). Permutation analysis revealed that the observed AUC was statistically significant (*p* < 0•05)

## 5 Discussion

Two findings stand out from our analysis. First, no alterations were observed in the candidate gene analysis, apart from a significant 1•3% decrease in methylation in the *CPT1A* gene in the FH mutation-negative group, suggesting that single gene methylation is not a cause of FH in our cohort. Secondly, gradient boosting machine learning revealed an overall difference in genome-wide DNA methylation between the FH mutation-positive and FH mutation-negative subjects, with a reasonable model performance (AUC 0•80±0•17). This finding underscores that these groups do differ from each other with regards to the epigenetic architecture at a genome-wide scale.

*CPT1A* was the only locus at which a statistical difference in methylation between the two groups was found. This gene encodes Carnitine palmitoyltransferase (*CPT1A*) and was found to be less methylated in FH mutation-negative patients compared to FH mutation-positive patients. CPT1A is a mitochondrial enzyme that catalyses the transfer of an acyl group from fatty acids to a carnitine molecule, hence controlling mitochondrial uptake and subsequent oxidation of the acyl group, especially in the liver. In line with this role in the regulation of fatty acid metabolism, hypomethylation of cg00574958 in the *CPT1A* gene is associated with plasma triglyceride concentrations [4,29]. However, in previous studies it has been shown that triglycerides affect methylation of *CPT1A* and not vice versa [30]. In fact, the observed lower cg00574958 methylation in the FH mutation-negative patients thus might be explained by the higher triglyceride levels in this group compared to the group of FH patients where a causative variant was identified (Table 1), since triglyceride levels were also found to negatively correlate with cg00574958 methylation in our study. Altogether, our results confirm the earlier described association between methylation in *CPT1A* and triglyceride levels, and a underlying mechanism of its relation to LDL cholesterol is likely not present and cannot be deducted from this study. Moreover, it is uncertain how a small methylation difference of 1.3% in this gene accounts for the severe hypercholesterolemic phenotype observed in the patients.

Next, we set out to incorporate methylation of CpG sites among the whole epigenome in a machine learning model to investigate whether the net effect of multiple small methylation differences could be used to identify specific patterns in FH mutation-negative and FH mutation-positive patients. Indeed, the resulting model performed well in distinguishing FH mutation-negative and FH mutation-positive patients (AUC 0•80±0•17), which emphasizes that the two selected FH groups differ on a genome-wide methylation level. The question arises whether the epigenetic changes in the group are causal or the consequence of environmental influences. For example, it might be that lifestyle factors resulting in triglyceride level differences between the two groups might also cause epigenetic difference, or that resulting triglycerides themselves influence genome wide methylation.

The top 20 CpG sites with a considerable impact on the model comprised two genes that have been linked to cholesterol metabolism in previous studies; *PRDM16* and *GSTT1. PRDM16* encodes PR/SET Domain 16, a protein involved in brown adipose tissue differentiation [31]. Common variants in the *PRDM16* locus are associated with plasma LDL-C and triglyceride levels [32], and methylation at CpG site cg26426080 is positively associated with *PRDM16* gene expression (Supplementary Table 3), suggesting that the observed hypomethylation in FH mutation-negative patients also reflects *PRDM16* expression differences in these patients. *GSTT1*, encoding Glutathione S-Transferase Theta 1, is an enzyme involved in the cellular defense against oxidative stress and genetic variants in this gene have been associated with risk for diabetes and atherosclerosis [33], and plasma total cholesterol, LDL-C and apolipoprotein B levels [34,35]. Like *PRDM16*, methylation of the identified CpG site in *GSTT1* (cg11478607) is correlated with expression of *GSTT1* (Supplementary Table 3), suggesting that the differential methylation observed in our study has an effect on *GSTT1* expression. However, the absolute differences in methylation in these two and the other top 20 CpG sites between the two groups is small (Supplementary Figure 2), suggesting that no single CpG methylation site is the causal factor for the phenotype in FH mutation-negative patients, but rather a result of the aggregate of a number of small methylation effects.

Our study has several limitations. Firstly, we measured DNA methylation in peripheral white blood cells, while the liver is known for its central role in LDL homeostasis. The results we obtained from the analyses in peripheral blood cells may therefore not reflect the deranged hepatic LDL metabolism in our patients. Secondly, the mutation-negative FH patient group comprised patients in whom not only epigenetic factors, but also other unknown genetic phenomena such as intronic variants [36] or polygenic hypercholesterolemia may be the causal factor [37]. Thirdly, as can be appreciated from Supplementary Figure 2, the machine learning model supposedly identified some CpG sites that had two or three distinguishable groups of methylation levels (e.g., *MYCBP*-cg24051749), suggesting the presence of a SNP despite the fact that we rigorously excluded CpG sites near SNPs according to the Illumina manifest using widely accepted pre-processing steps before the analysis. The used gradient boosting model, however, allows for the identification of DNA methylation differences between the two groups despite the presence of skewed distributed methylation data because of a SNP. Further studies should be executed to assess whether the SNP has biological relevant effects in these patients or that they are coincidently identified. Moreover, in our study the group of FH mutation-negative patients were diagnosed with FH by the referring physician based on national guidelines [12,13] and thus potentially is a non-homogenous clinical FH group characterized by some characteristic differences with the FH mutation-positive patients. For example, the FH patients with a *LDLR* pathogenic variant were younger and the LDL-C levels were higher compared to FH variant negative patients (Table 1). Although age and lipoproteins can modulate DNA methylation [30], we estimate this effect to be minimal since we explored methylation only in patients with very high LDL-C levels (above 6 mmol/L and above the 99th percentile in the general population) in both groups. Furthermore, we selected only male participants who were not using statins, since these lipid lowering drugs have been shown to alter DNA methylation through reducing DNA methyltransferase mRNA levels [38], and are associated with less methylation in promotor regions of various genes [39,40]. It is also possible that other confounders, such as obesity, are present in the current study. Additionally, we enrolled a relatively small number of individuals in our study. Our stringent selection criteria to avoid spurious findings did not allow for a larger study group to be analysed. Lastly, in the current model we analysed the data at a group level, and we might therefore have missed specific causal methylation patterns that would explain the FH phenotype at an individual patient level.

Despite extensive sequencing efforts, a causative genetic variant is not found in a large proportion of patients with a clinical FH diagnosis [1]. Hence efforts to find novel factors causing the FH phenotype are deemed of great relevance. The data presented in the current study suggest that monogenic DNA methylation alterations are not a major contributing factor in FH in our cohort and thus are unlikely to be a common contributing factor to the FH phenotype in FH mutation-negative patients. Nevertheless, with the current study we have not excluded the possibility that rare monogenic DNA methylation alterations can cause FH in some individuals. On the other hand, the genome-wide methylation differences observed with advanced machine learning models between FH mutation-negative and FH mutation-positive subjects might suggest that a large number of small DNA methylation effects play a role in high plasma LDL-C. This phenomenon resembles the polygenic score where the inter individual differences in LDL-C levels are not explained by individual genetic variations but rather by the sum of a large number of small effect-size genetic factors. The question whether this is clinically relevant ensues from this finding. In contrast to monogenic FH, family screening for the presence of polygenic hypercholesterolemia, and epigenetic hypercholesterolemia, does not make sense as these do not follow an autosomal dominant inheritance pattern. At this stage, the treatment of these patients will not change either, since FH guidelines recommend the same aggressive lipid lowering with statins and add-on therapeutics, irrespective of the FH cause. Epigenetic hypercholesterolemia may only prove to be clinically relevant in case it has an impact on the efficacy of lipid lowering therapies.

This study was the first of its kind to be conducted in FH patients and tried to control for confounding by differences in lipid levels by the inclusion of two unique FH patient groups: those of interests, FH mutation-negative patients, and a group of FH mutation-positive patients. Although classical candidate gene analysis did, except for *CPT1A*, not reveal major DNA methylation differences in known lipid genes, a machine learning approach showed that FH mutation-negative patients are characterized by a different genome wide DNA methylation pattern compared to FH mutation-positive patients, with important model features for the genes *PRDM16* and *GSTT1*.

*Data sharing statement:* All individual normalized DNA methylation data are available via https://dx.doi.org/10.6084/m9.figshare.12334586.

## Funding sources

This study was funded by a 10.13039/501100001826ZonMW grant (VIDI No. 016.156.445) obtained by G.K. Hovingh. The funder (ZonMW) was not involved in the design, data collection, analysis, interpretation or any other aspect of this study.

## Author contributions

Conceptualization, L.F.R., P.H., and G.K.H.;

Methodology, L.F.R., E.L., A.K.G., P.H. and G.K.H.;

Formal Analysis, L.F.R., A.V. and J.P.B.P.;

Resources, J.C.D. and G.K.H.;

Writing – Original Draft, L.F.R.;

Writing-Review & Editing, E.L., M.N., A.K.G., A.G., P.H. and G.K.H.;

Visualization, L.F.R., J.P.B.P. and L.E.

Supervision, P.H. and G.K.H.;

Funding Acquisition, G.K.H.

## Declaration of Competing Interests

LFR is co-founder of Lipid Tools B.V. MN reports reimbursement from kaleido biosciences and caelus health, outside the submitted work. GKH has served as consultant and speaker for biotechnology and pharmaceutical companies that develop molecules that influence lipoprotein metabolism, including Regeneron, Aegerion Pfizer, Merck, KOWA, Sanofi, and Amgen; has served as principal investigator for clinical trials conducted with a.o. Amgen, Sanofi, Eli Lilly, Novartis, Kowa, Genzyme, Cerenis, Pfizer, Dezima, and AstraZeneca; has received research grants from ZonMW (Vidi grant [016.156.445]), Klinkerpad fonds, the European Union, Amgen, Sanofi, AstraZeneca, Aegerion, and Synageva; has received honoraria and investigator fees (to the Department of Vascular Medicine) for sponsor-driven studies and lectures for companies with approved lipid-lowering therapy in the Netherlands; and is partly employed by Novo Nordisk AS, Copenhagen, Denmark (0.7FTE) and the Amsterdam UMC, Amsterdam, the Netherlands (0.3FTE). All other authors declare no competing interests.

## Appendix Supplementary materials

Image, application 1
