# Supplementary material for: Differential DNA methylation in familial hypercholesterolemia
Source: eBioMedicine. 2020 Oct 21;61:103079. doi: 10.1016/j.ebiom.2020.103079 (PMC7581877; doi:10.1016/j.ebiom.2020.103079)

**SUPPLEMENTAL MATERIALS**

**Differential DNA methylation in familial hypercholesterolemia**

Laurens F. Reeskamp, Andrea Venema*, Joao P. Belo Pereira*, Evgeni Levin, Max Nieuwdorp, Albert K. Groen, Joep C. Defesche, Aldo Grefhorst, Peter Henneman, G. Kees Hovingh

Supplementary Tables:

1. Candidate gene tiers
2. CpG sites with >10% relative feature importance in machine learning model
3. Association between identified CpG sites of interest methylation and expression in public databases
4. Next-generation sequencing gene panel

Supplementary Figures:

1. Inflation correction with BACON package of candidate gene analysis
2. Methylation of the top 20 individual CpG sites derived from the machine learning model
3. Feature importance of top 20 features in machine learning model

**Supplementary Table 1: Candidate gene tiers**

| **Tier** | **Gene** |
| --- | --- |
| 1 | *LDLR* |
| 1 | *APOB* |
| 1 | *PCSK9* |
| 2 | *LDLRAP1* |
| 2 | *STAP1* |
| 2 | *ABCG5* |
| 2 | *ABCG8* |
| 2 | *APOE* |
| 2 | *LIPA* |
| 3 | *ANXA9* |
| 3 | *CERS2* |
| 3 | *EHBP1* |
| 3 | *BRCA2* |
| 3 | *FN1* |
| 3 | *APOH* |
| 3 | *PRKCA* |
| 3 | *SPTLC3* |
| 3 | *SNX5* |
| 3 | *MTMR3* |
| 3 | *NYNRIN* |
| 3 | *INSIG2* |
| 3 | *LINC01101* |
| 3 | *CMTM6* |
| 3 | *CSNK1G3* |
| 3 | *SOX17* |
| 3 | *UGT1A1* |
| 3 | *VLDLR* |
| 3 | *DLG4* |
| 3 | *PRARA* |
| 3 | *SORT1* |
| 3 | *MYLIP* |
| 3 | *HFE* |
| 3 | *LPA* |
| 3 | *PLEC1* |
| 3 | *ABO* |
| 3 | *ST3GAL4* |
| 3 | *OSBPL7* |
| 3 | *TOP1* |
| 3 | *MARC1* |
| 3 | *IRF2BP2* |
| 3 | *HMGCR* |
| 3 | *HLA-DRA* |
| 3 | *FRK* |
| 3 | *DNAH11* |
| 3 | *NPC1L1* |
| 3 | *CYP7A1* |
| 3 | *GPAM* |
| 3 | *BRAP* |
| 3 | *HNF1A* |
| 3 | *HPR* |
| 3 | *MAFB* |
| 3 | *ANGPTL3* |
| 3 | *MIR148A* |
| 3 | *LRPAP1* |
| 3 | *TIMD4* |
| 3 | *CILP2* |
| 3 | *ASAP3* |
| 3 | *ABCB11* |
| 3 | *FAM117B* |
| 3 | *PXK* |
| 3 | *KCNK17* |
| 3 | *HBS1L* |
| 3 | *GPR146* |
| 3 | *VIM* |
| 3 | *CUBN* |
| 3 | *PHLDB1* |
| 3 | *PHC1* |
| 3 | *A2ML1* |
| 3 | *TOM1* |
| 3 | *EVI5* |
| 3 | *RAB3GAP1* |
| 3 | *RAF1* |
| 3 | *C6orf106* |
| 3 | *SPTY2D1* |
| 3 | *MAMSTR* |
| 3 | *ERGIC3* |
| 3 | *PPP1R3B* |
| 3 | *APOE* |
| 3 | *CETP* |
| 3 | *TRIB1* |
| 3 | *FADS1* |
| 3 | *FADS2* |
| 3 | *FADS3* |
| 3 | *APOA1* |
| 3 | *PIGV* |
| 3 | *NR0B2* |
| 3 | *ACAD11* |
| 4 | *DHCR24* - cg17901584 |
| 4 | *OXER1* - cg23759710 |
| 4 | *SQLE* - cg00285394 |
| 4 | *NLRC5* - cg00285394 |
| 4 | *SREBF2* - cg09978077 |
| 4 | *TNIP1* - cg22178392 |
| 4 | *STAT5A* - cg03001305 |
| 4 | *CPT1A* - cg00574958 |
| 4 | *PTAFR* - cg20460771 |
| 4 | *IL6R* - cg09257526 |
| 4 | *ACP1* - cg05464506 |
| 4 | *CIAO1* - cg25522181 |
| 4 | *FYN* - cg08376209 |
| 4 | *ARID3B* - cg02384859 |
| 4 | *ARHGEF1* - cg12168357 |
| 4 | *DEDD2* - cg13790259 |
| 4 | *APOBEC3H* - cg06229674 |
| 4 | *ARHGEF9* - cg00369058 |

**Supplementary Table 2 CpG sites with >10% relative feature importance in machine learning model**

| **CpG** | **Gene** | **Chromosome** | **Position** | **Relative Feature Importance** |
| --- | --- | --- | --- | --- |
| cg14265823 | *PAX3* | chr2 | 223163326 | 100 |
| cg02558132 | *MYLK* | chr3 | 123411198 | 97.97 |
| cg22162835 | *TEAD3* | chr6 | 35457472 | 92.2 |
| cg00415024 |  | chr20 | 56044352 | 87.39 |
| cg26426080 | *PRDM16* | chr1 | 3039210 | 84.61 |
| cg07051648 | *NTN5/SEC1P* | chr19 | 49177693 | 76.65 |
| cg05071823 | *DOCK11* | chrX | 117628671 | 61.17 |
| cg05541727 | *EXD3* | chr9 | 140277740 | 54.31 |
| cg24051749 | *MYCBP* | chr1 | 39340282 | 53.71 |
| cg11478607 | *GSTT1* | chr22 | 24384400 | 51.79 |
| cg10020385 | *MAF1* | chr8 | 145159706 | 49.8 |
| cg11136235 |  | chr10 | 81077552 | 48.55 |
| cg16370685 | *SETDB1* | chr1 | 150899163 | 46.59 |
| cg09138267 | *LOC728743* | chr7 | 150102791 | 46.47 |
| cg04900489 |  | chr13 | 31272551 | 46.29 |
| cg16685760 |  | chrX | 145701257 | 46.17 |
| cg07336544 | *KCNMA1* | chr10 | 79194347 | 44.54 |
| cg00578917 | *CYYR1* | chr21 | 27945542 | 42.69 |
| cg20588438 | *KNTC1* | chr12 | 123089881 | 41.65 |
| cg15458017 |  | chr17 | 9672274 | 41.5 |
| cg21166457 |  | chr14 | 106539756 | 41.34 |
| cg23350716 | *PPIAL4B* | chr1 | 147956744 | 40.89 |
| cg17251658 | *CBLN1* | chr16 | 49315754 | 40.29 |
| cg14705391 |  | chr2 | 16161202 | 39.64 |
| cg07052624 | *ZNF565* | chr19 | 36706445 | 38.29 |
| cg26111308 | *TVP23B* | chr17 | 18699050 | 38.25 |
| cg09661370 | *HOXA11AS* | chr7 | 27225299 | 38.02 |
| cg04515200 |  | chr5 | 135415762 | 37.1 |
| cg00828689 | *PIGZ* | chr3 | 196697064 | 36.07 |
| cg09049879 | *TEX30* | chr13 | 103423502 | 36.03 |
| cg22867893 | *ARRB1* | chr11 | 74988026 | 34.02 |
| cg12739319 | *INSL6* | chr9 | 5186766 | 32.06 |
| cg10207850 |  | chr2 | 26955177 | 32.04 |
| cg19384241 |  | chr2 | 55393977 | 31.58 |
| cg20758756 |  | chr18 | 9321560 | 31 |
| cg04497520 | *PHF13* | chr1 | 6673429 | 30.44 |
| cg07158503 |  | chr5 | 135415693 | 29.79 |
| cg14755254 | *ERICH1* | chr8 | 637813 | 29.18 |
| cg03515999 | *PSMD5* | chr9 | 123606332 | 28.72 |
| cg18091165 | *BAT1* | chr6 | 31509352 | 27.93 |
| cg12367543 |  | chr22 | 48602194 | 27.92 |
| cg06489993 | *PPP5D1* | chr19 | 47082996 | 25.88 |
| cg20707527 |  | chr8 | 105343122 | 25.85 |
| cg25374269 |  | chr13 | 44887281 | 25.71 |
| cg22713356 |  | chr15 | 30763199 | 24.55 |
| cg02333792 | *RASGRF1* | chr15 | 79269735 | 24.14 |
| cg27219185 |  | chr20 | 11360021 | 24.1 |
| cg03881738 |  | chr6 | 170547103 | 24.08 |
| cg27586797 |  | chr5 | 13664584 | 23.66 |
| cg05745656 |  | chr6 | 22271391 | 23.34 |
| cg19872188 | *LOC285740* | chr6 | 143884511 | 23.08 |
| cg22138327 | *GTF3A* | chr13 | 27999177 | 22.59 |
| cg05194426 | *CYP2E1* | chr10 | 135343193 | 21.64 |
| cg16604835 | *NRCAM* | chr7 | 108043396 | 21.63 |
| cg26999053 | *MYH10* | chr17 | 8414418 | 20.92 |
| cg14614539 | *NOTCH4* | chr6 | 32170458 | 20.87 |
| cg01053087 | *ERICH1* | chr8 | 637909 | 20.78 |
| cg01519350 | *ARMC8* | chr3 | 137906342 | 20.26 |
| cg04255814 | *LINC00222* | chr6 | 109072980 | 20.24 |
| cg09125754 | *POTEF* | chr2 | 130886714 | 19.81 |
| cg14378789 |  | chr2 | 112483666 | 19.68 |
| cg07629776 | *FRMD4A* | chr10 | 13972210 | 19.61 |
| cg08159444 | *PNMA5* | chrX | 152160229 | 19.53 |
| cg10667338 | *LIN7B* | chr19 | 49617042 | 18.28 |
| cg12002139 | *SYNJ2* | chr6 | 158478872 | 17.73 |
| cg20432732 | *UBR4* | chr1 | 19451037 | 17.63 |
| cg17662387 | *ABLIM1* | chr10 | 116286880 | 17.34 |
| cg09506600 | *OR2L13* | chr1 | 248100228 | 17.3 |
| cg18434912 | *AMD1* | chr6 | 111194786 | 17.21 |
| cg20227896 | *SPTBN4* | chr19 | 41069670 | 17.14 |
| cg13984289 | *MYH13* | chr17 | 10220829 | 17.13 |
| cg11787167 | *NPAS3* | chr14 | 33407370 | 17.09 |
| cg12718519 | *PRKCZ* | chr1 | 2058417 | 17.04 |
| cg21754534 | *DGKD* | chr2 | 234294601 | 16.78 |
| cg20787649 | *PADI4* | chr1 | 17636898 | 16.67 |
| cg06069504 |  | chr19 | 10707453 | 16.44 |
| cg19469447 | *CYP2E1* | chr10 | 135341870 | 16.41 |
| cg25533845 | *MTRF1* | chr13 | 41838126 | 16.38 |
| cg21415084 |  | chr12 | 84218134 | 16.17 |
| cg08291957 | *P4HA1* | chr10 | 74821035 | 16.13 |
| cg09723679 | *RNF145* | chr5 | 158634676 | 16.06 |
| cg07167872 | *PM20D1* | chr1 | 205819463 | 16.04 |
| cg08164151 |  | chr12 | 131118432 | 15.97 |
| cg10980791 | *FAM155A* | chr13 | 108118957 | 15.94 |
| cg02087342 | *TP53* | chr17 | 7579047 | 15.86 |
| cg02673002 | *RNF169* | chr11 | 74459317 | 15.75 |
| cg10154826 | *FAM8A1* | chr6 | 17600994 | 15.64 |
| cg20347269 | *P2RX1* | chr17 | 3808157 | 15.64 |
| cg19850149 |  | chr5 | 180397496 | 15.45 |
| cg04898932 |  | chr22 | 35645717 | 15.36 |
| cg11261447 | *NLRP1* | chr17 | 5465054 | 15.3 |
| cg09840753 | *WDR25* | chr14 | 100844352 | 15.18 |
| cg26491092 |  | chr1 | 23510886 | 15.04 |
| cg17190362 | *LAMA4* | chr6 | 112475675 | 14.86 |
| cg06427702 | *PPAPDC1A* | chr10 | 122228021 | 14.84 |
| cg14398247 | *ARFGAP3* | chr22 | 43216462 | 14.83 |
| cg08299859 |  | chr10 | 13259274 | 14.73 |
| ch.7.770055F |  | chr7 | 31513616 | 14.68 |
| ch.8.1073630F | *KIAA0146* | chr8 | 48637817 | 14.65 |
| cg11878555 |  | chr1 | 31920316 | 14.64 |
| cg03485872 |  | chr4 | 127657778 | 14.59 |
| cg13033971 |  | chr13 | 46291973 | 14.27 |
| cg14331362 |  | chr2 | 242948396 | 14.21 |
| cg27629640 | *ALOXE3* | chr17 | 8022337 | 14.18 |
| cg13023205 |  | chr17 | 81060243 | 14.13 |
| cg06344265 | *GRIK4* | chr11 | 120530973 | 13.95 |
| cg25420767 | *ABLIM3* | chr5 | 148620091 | 13.56 |
| cg12067536 | *ARHGAP42* | chr11 | 100558822 | 13.54 |
| cg10096454 | *LDB1* | chr10 | 103880868 | 13.15 |
| cg06675417 |  | chr18 | 77292443 | 13.13 |
| cg18792536 | *UPK3B* | chr7 | 76145562 | 13.11 |
| cg09296044 | *CCNDBP1* | chr15 | 43477606 | 13.08 |
| cg06734157 |  | chr3 | 150066462 | 13.03 |
| cg16079430 |  | chr5 | 10856757 | 12.95 |
| cg27281559 |  | chrX | 145701263 | 12.94 |
| cg21240272 | *NXNL2* | chr9 | 91174472 | 12.84 |
| cg19090364 | *ITCH* | chr20 | 32972071 | 12.83 |
| cg01451203 | *CYFIP1* | chr15 | 22955743 | 12.82 |
| cg02352685 | *BTNL8* | chr5 | 180377617 | 12.76 |
| cg14554157 | *BCL11A* | chr2 | 60780027 | 12.75 |
| cg17215819 | *LOC101927502* | chr9 | 84386662 | 12.71 |
| cg21149944 | *ZNF718* | chr4 | 124344 | 12.67 |
| cg01780345 | *CFAP58* | chr10 | 106147097 | 12.56 |
| cg13691793 | *OSBPL1A* | chr18 | 21750891 | 12.51 |
| cg14353184 |  | chr6 | 32584893 | 12.46 |
| cg22730047 |  | chr1 | 161410551 | 12.34 |
| cg26725559 |  | chr18 | 9321178 | 11.98 |
| cg15098922 |  | chrX | 114579157 | 11.97 |
| cg03966322 | *NADK2* | chr5 | 36242514 | 11.87 |
| cg04221355 | *BRE* | chr2 | 28114116 | 11.65 |
| cg25880954 | *MGC12982* | chr1 | 47900630 | 11.63 |
| cg19347782 | *MICAL2* | chr11 | 12159762 | 11.63 |
| cg09062922 |  | chr5 | 131436890 | 11.6 |
| cg11186706 |  | chr14 | 54815745 | 11.43 |
| cg02331830 | *PLEC1* | chr8 | 145008288 | 11.41 |
| cg12628883 | *PRTFDC1* | chr10 | 25219837 | 11.39 |
| cg20517941 | *LOC100507140* | chr2 | 201600636 | 11.16 |
| cg02484732 | *COL21A1* | chr6 | 56096198 | 11.06 |
| cg17460047 | *ZNRF2* | chr7 | 30323918 | 10.97 |
| cg07796016 | *LCE1C* | chr1 | 152779584 | 10.84 |
| cg16106431 | *ABLIM2* | chr4 | 8034335 | 10.84 |
| cg10681804 | *GGT7* | chr20 | 33433114 | 10.82 |
| cg11304899 | *LINC01055* | chr13 | 46267080 | 10.8 |
| cg07504457 | *VPS28* | chr8 | 145652410 | 10.78 |
| cg11688874 | *WAC* | chr10 | 28822482 | 10.77 |
| cg09015484 |  | chr9 | 96929106 | 10.73 |
| cg16511841 | *C6orf167* | chr6 | 97730490 | 10.7 |
| cg21635219 | *ACSL4* | chrX | 108973840 | 10.61 |
| cg16145915 | *ZFAND2A* | chr7 | 1198662 | 10.57 |
| cg00013655 | *PIGQ* | chr16 | 629015 | 10.49 |
| cg10625758 | *WBP2* | chr17 | 73851514 | 10.46 |
| cg20792978 | *ZDHHC9* | chrX | 128977934 | 10.36 |
| cg01462207 | *POLE* | chr12 | 133202012 | 10.3 |
| cg19437126 |  | chr12 | 91720401 | 10.24 |
| cg05571310 | *KIF19* | chr17 | 72350354 | 10.1 |

CpG sites, their respective linked genes and genomic positions, that are most predictive for distinguishing FH mutation-positive and FH mutation-negative patients based on the machine learning model measured as relative feature importance. Only CpG sites with a relative feature importance >10% are reported. Genomic positions as provided in human genome build – hg19.

**Supplemental Table 3: Association between identified CpG sites of interest methylation and expression in public databases**

|  | **SMART** | | **MEXPRESS** | |
| --- | --- | --- | --- | --- |
| **CpG** | **R*** | **P-value** | **R**** | **P-value** |
| *Candidate Gene Analysis* | | | | |
| *CPT1A*- cg00574958 | -0.083 | 0.093 | -0.198 | <0.001 |
| *Top Features Machine Learning Model* | | | | |
| *PAX3*-cg14265823 | -0.12 | 0.019 | -0.0862 | ≥0.05 |
| *MYLK*-cg02558132 | 0.019 | 0.71 | 0.0732 | ≥0.05 |
| *PRDM16*-cg26426080 | 0.38 | 1.5 x 10^-15^ | 0.496 | <0.001 |
| *NTN5*-cg07051648 | 0.071 | 0.15 | NA | NA |
| *DOCK11*-cg05071823 | 0.029 | 0.56 | 0.0971 | ≥0.05 |
| *MYCBP*-cg24051749 | NA | NA | NA | NA |
| *GSTT1*-cg11478607 | NA | NA | -0.876 | <0.001 |
| cg11136235 | NA | NA | NA | NA |
| *SETDB1*-cg16370685 | -0.26 | 2.2 x 10^-7^ | -0.157 | <0.01 |
| *LOC728743*-cg09138267 | NA | NA | NA | NA |
| cg16685760 | NA | NA | NA | NA |

Correlation and P-values for CpG methylation and expression were derived from two publicly accessible databases for tumor specific tissues (SMART [Li 2019] and MEXPRESS [Koch 2015]). The reported correlation was derived from liver hepatocellular carcinoma(LIHC) subset. Since these two databases only contain CpG sites that are available on the 450k Illumina Infinium beachip assay, only those CpG sites are reported in this table.
* Spearman rank correlation
** Pearson correlation

**Supplementary Table 4: Next-generation sequencing gene panel**

| Gene |
| --- |
| *LDLR* |
| *APOB* |
| *PCSK9* |
| *LIPA* |
| *LDLRAP1* |
| *ABCG5* |
| *ABCG8* |
| *STAP1* |
| *ANGPTL3* |
| *MTTP* |
| *MYLIP* |
| *ABCA1* |
| *LCAT* |
| *APOA1* |
| *SCARB1* |
| *CETP* |
| *LIPG* |
| *LIPC* |
| *APOC3* |
| *LPL* |
| *APOC2* |
| *APOA5* |
| *GPIHBP1* |
| *LMF1* |
| *APOE* |
| *SAR1B* |
| *CYP27A1* |

**Supplementary Figure 1: Inflation correction with BACON package of candidate gene analysis**


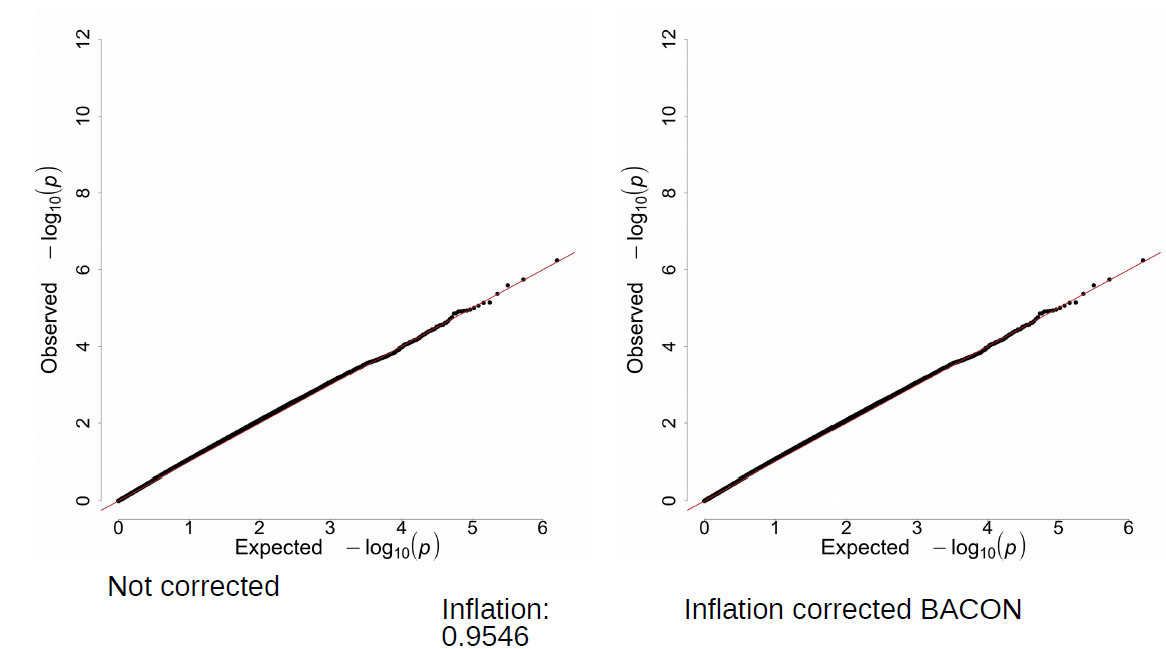


**Supplementary Figure 2: Methylation of the top 20 individual CpG sites derived from the machine learning model**


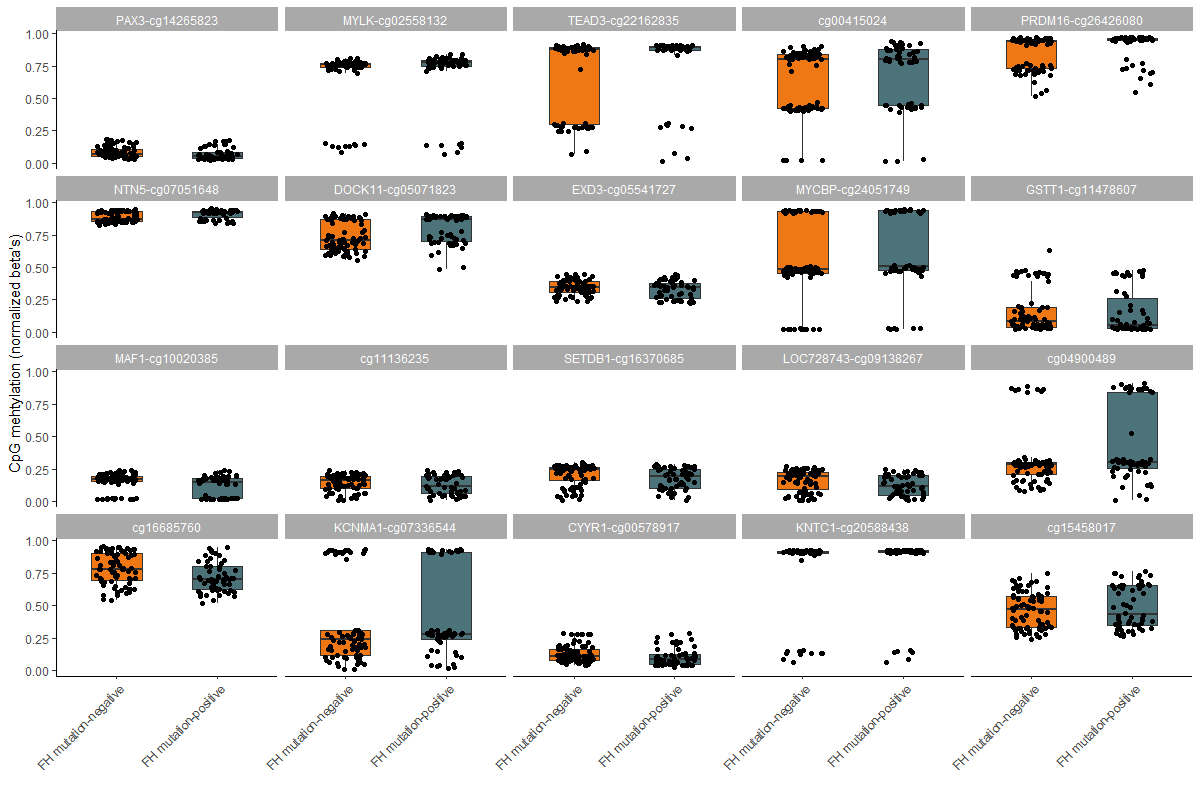

Supplement: Supplementary file 1 [file mmc1.docx]
